# Acceptance of the COVID-19 vaccine booster dose and associated factors among the elderly in China based on the health belief model (HBM): A national cross-sectional study

**DOI:** 10.3389/fpubh.2022.986916

**Published:** 2022-12-15

**Authors:** Chenyuan Qin, Wenxin Yan, Min Du, Qiao Liu, Liyuan Tao, Min Liu, Jue Liu

**Affiliations:** ^1^Department of Epidemiology and Biostatistics, School of Public Health, Peking University, Beijing, China; ^2^Research Center of Clinical Epidemiology, Peking University Third Hospital, Beijing, China; ^3^Institute for Global Health and Development, Peking University, Beijing, China; ^4^National Health Commission Key Laboratory of Reproductive Health, Peking University, Beijing, China

**Keywords:** COVID-19, vaccination, booster dose, hesitancy, old people

## Abstract

**Background:**

The reluctance of individuals to obtain solid vaccine-induced immunity represents a fundamental challenge to containing the spread of SARS-CoV-2, including its highly mutated variants. We aimed to assess vaccination acceptance and associated factors for the COVID-19 vaccine booster dose among elderly people (≥60 years old) in China, providing a theoretical and practical reference for universal vaccination policy.

**Methods:**

A national anonymous survey was conducted in mainland China from May 25 to June 8, 2022, using a stratified random sampling method. Individuals 60 years of age and above were the target population. A chi-squared test and Cochran-Armitage test for trend were used to compare and examine vaccine acceptance rates by characteristics. *Via* a backward stepwise method, multivariable logistic regression models were established to assess factors associated with booster dose acceptance. Two-sided *P* < 0.05 was considered statistically significant.

**Results:**

Of 3,321 eligible participants, 82.8% (95% CI: 81.5–84.1%) were willing to receive COVID-19 vaccine booster shots. Concerns about contraindications (38.3%), vaccine safety (32.0%), and limited movement (28.0%) were the main reasons for vaccine hesitancy. Nearly one-third still believed that the booster dose was unnecessary after receiving the initial vaccination. Older adults with a low level of perceived barriers (aOR = 1.86, 95% CI, 1.03–3.38), a high level of perceived benefit (aOR = 2.31, 95% CI, 1.38–3.87), and higher cues to action (moderate, aOR = 2.22, 95% CI, 1.39–3.56; high, aOR = 5.46, 95% CI: 3.44–8.67) were more likely to accept the booster dose. Other major factors affecting the booster dose acceptance rate were occupation, time spent on social media, vaccination history, and a high knowledge score for COVID-19 and vaccines. In addition, for those over 70 years of age, rising awareness of susceptibility could be a better gateway for improving their willingness to get vaccinated.

**Conclusions:**

A total of 82.8% of recruited older adults were willing to receive the booster dose. Acceptance behaviors were closely related to occupation, time spent on social media, vaccination history, knowledge factors, perception of barriers, and benefit, as well as action cues. Targeted public health measures are a priority for improving the vaccination coverage of valid immunity among the elderly population, not only to prevent infection and poor prognosis caused by emerging variants but also to reduce the huge disease and economic burden caused by the long-term sequelae after SARS-CoV-2 infection.

## Introduction

As a salient example of an infectious disease with profound economic, social, and health implications, coronavirus disease 2019 (COVID-19) has directly affected the health of 628.6 million people as of October 26, 2022, and has caused 6.6 million deaths (1, 2). Severe acute respiratory syndrome coronavirus 2 (SARS-CoV-2) changes over time, and Omicron (B.1.1.529) is the dominant variant at the time of publication (3, 4). With multiple spike mutations and high transmissibility and immune escape, it outcompeted former variants soon after it was first detected in November 2021 (3, 4). Undoubtedly, promoting the global uptake of initial COVID-19 vaccines and booster doses has become an urgent issue worldwide, and is closely related to the formation of herd immunity (5, 6). According to data released by the United Nations Population Division, the global population aged 60 years and above was 1.08 billion in 2021, accounting for 13.7% of the global population (7). The elderly population is a key factor in the fight against COVID-19. Recently, BA.4 and BA.5 subvariants have emerged as the dominant strains and have higher transmissibility and immune escape ability than previous Omicron subvariants (8). Given the high risk of contracting SARS-CoV-2 and poor prognosis, older adults are confronted by the need to achieve solid immunity *via* the initial COVID-19 vaccine or the booster dose (9, 10). Moreover, a recent cohort study suggested that older age and multiple underlying diseases were risk factors for the development of long COVID-19, while valid vaccination may reduce the chance of long-term sequelae (11).

The reluctance of people to obtain solid vaccine-induced immunity represents a fundamental challenge to containing the spread of SARS-CoV-2 (12, 13). According to a report about the fifth wave of COVID-19 in Hong Kong, China, people aged 60 or above accounted for 96% of all deaths, and 88% of the deaths were people that were either unvaccinated or received only one dose of COVID-19 vaccine (14). However, only 32.3% of the world's population has received booster doses against COVID-19, as of October 8, 2022 (15). The Joint Prevention and Control Mechanism of the State Council claimed that, as of November 4, 2022, 31.6% of the elderly (≥60 years old) in China have yet to receive booster shots (16). Another critical issue that needs to be highlighted is that the overall level of effective antibodies produced by older people after vaccination was lower than that produced by younger adults (10, 17). Obviously, identifying misperceptions that fuel vaccine hesitancy and creating effective communication strategies are global public health priorities (12, 13, 18). The health belief model (HBM) is a health education model that can change people's behavior by intervening in people's perception, attitude, and belief (19–21). Individual health beliefs, cues to action, and behavioral constraints (demographic characteristics, knowledge level, etc.) constitute a complete HBM (22–24). Individual health belief refers to an individual's thoughts on health and disease, including recognizing the severity of the disease and their susceptibility (perceived severity and susceptibility), understanding the benefit of taking preventive measures and obstacles in the process (perceived benefit and barriers), and an individual's judgment about his ability to perform a certain action (self-efficacy). Action cues refer to factors that prompt people to take preventive measures, including reminders from medical staff. Combining motivation theory, cognitive theory, and expectancy-value theory, the HBM was used to predict people's preventive health behaviors and implement health education (25, 26). In recent years, HBM has been widely used to predict and explain the acceptance of vaccines against COVID-19, measles, human papillomavirus, etc., among different populations and its influencing factors (27–32). Combining the findings across studies, people with high perceived susceptibility, high perceived severity, high perceived benefit, and high levels of action cues were more likely to get vaccinated. The level of perceived barriers was generally inversely related to vaccination intention (27–32).

The entire medical community has invested enormous efforts in developing and delivering COVID-19 vaccines, and vaccination campaigns among adults are in full swing, such as in China. The Chinese government has continued to encourage older people to get vaccinated, especially for booster shots (33, 34). Communities have also launched a series of campaigns to increase vaccination rates among older people (33–35). Given the current more infectious variant, decreased vaccine-induced antibody titers, and the special social status of elderly individuals, vaccination intention and influencing factors of the elderly were essential for promoting vaccination coverage. However, few studies have targeted the elderly population (≥60 years old). Although our previous study shows that 93.7% of Chinese adults were willing to receive a third dose, only 81.7% (67/82) of people over the age of 50 have exhibited a willingness to take the vaccine (19). Obviously, owing to the small sample size of elderly people, previous results are of limited significance and are not sufficient to guide practical efforts for improving vaccine coverage in the elderly population. Therefore, we conducted a new survey based on the original study and targeted people aged 60 and above in China with the aim of identifying factors that can fuel vaccine acceptance and provide theoretical and practical references for universal vaccination policy.

## Methods

### Study design, population, and sampling

This national anonymous cross-sectional survey was conducted in mainland China *via* an online platform called Wen Juan Xing (Changsha Ranxing Information Technology Co., Ltd., Hunan, China). Covering at least 2.6 million registered members in China, we could easily reach an authentic, diverse, and representative sample (21, 36). Questionnaires were distributed *via* the internet to the potential target groups in the sample bank of this professional data science company. Our inclusion criteria were as follows: (1) Chinese citizens; (2) ≥60 years old; (3) people who agreed to participate in this survey; and (4) completed the survey between May 25, 2022 and June 8, 2022. To recruit enough representative respondents, we randomly sampled potentially eligible subjects in 31 provinces according to the proportion of adults aged 60 and above reported in the Seventh National Census (37). For people who did not respond to the questionnaire, the platform conducted further random sampling and sent questionnaires according to the preset requirements, ensuring that sufficient and high-quality samples were obtained.

Integrating the findings of previous studies and expert opinions, we set the rate of COVID-19 vaccine booster acceptance as 87% (*P* = 0.87) among older people (38, 39). PASS software 15.0 (NCSS LLC., Kaysville, U.T., USA) was used to calculate the minimum sample size with an acceptance rate of 87%, an α of 0.05, and a two-sided confidence interval width of 0.2p (0.174), using the exact (Clopper-Pearson) method. After quality control and manual check procedures to exclude invalid questionnaires, a total of 3,321 eligible respondents were ultimately included in our analyses (Supplementary Table S1).

### Questionnaire design

The questionnaire was constructed and revised by a panel of experts, including one public health expert and two epidemiologists specializing in infectious diseases (Supplementary material 2). We performed a presurvey before it was officially released to test the validity of the questionnaire (Bartlett's test of sphericity, *P* < 0.001). The reliability of this questionnaire (only for the scales in the questionnaire) was confirmed by Cronbach's alpha coefficient by different dimensions (all α > 0.75). Each question was followed by a reminder that all answers were supposed to reflect the reality and true thoughts of those older adults.

The primary outcome was the acceptance rate of the COVID-19 vaccine booster, defined as the proportion of participants who answered “yes” when asked whether they were willing to receive the COVID-19 vaccine booster dose if available. If a respondent had any concerns or reluctance, the questionnaire would automatically jump to the specific reasons for hesitation.

Sociodemographic characteristics and health status of the sample population were investigated, including region, age, sex, marital status, education level, occupation, income, history of chronic disease, and COVID-19 vaccination. The amount of time people spent checking the news about COVID-19 or vaccines on social media each day and people's satisfaction with the government's response to COVID-19 were also obtained.

For knowledge factors, we set six and four questions to determine the knowledge level of the elderly on COVID-19 and COVID-19 vaccines, respectively. Sources of SARS-CoV-2 infection, common symptoms, transmission routes, high-risk groups, self-protection measures, and herd susceptibility were the investigational scope of the former aspect, with a total of 19 scores. Scores of “0–6,” “7–13,” and “14–19” represented a “low,” “moderate,” and “high” degree, respectively. For the knowledge on COVID-19 vaccines component, we mainly focused on price, time for booster vaccination, immunity induced by vaccines, and adverse reactions after vaccination. Each correct choice received 1 point, and a total of four scores were assigned to this part. Then, all respondents were divided into three groups (score 0–1, 2–3, and 4) from low to high.

As one of the most common theories reflecting individual behavior change, the HBM is an organic combination of motivation theory, cognitive theory, and expectancy-value theory, which has been widely used to explain public attitudes toward vaccines and predict people's vaccination behavior (19–21). Considering the strong correlation between different factors of the original HBM, “self-efficacy” was not included in the adjusted HBM of our study. We chose five dimensions in our final questionnaire, including perceived susceptibility (two items), perceived severity (two items), perceived barriers (three items), perceived benefit (three items), and cues to action (three items). All questions were answered as either “very concerned/agree,” “concerned/not sure,” and “not concerned/disagree” through a three-point Likert scale, and scores of 3, 2, and 1 were assigned to the above answers in descending order. Finally, an individual's grade in each dimension was divided into “low,” “moderate,” and “high” in ascending order based on their total points.

### Data analysis

We performed descriptive analyses to summarize the characteristics of the recruited population by frequencies and percentages. The independent Chi-squared test and Cochran-Armitage test for trend were used to compare the differences between groups stratified by the abovementioned characteristics. To identify factors that influence vaccination willingness, multivariable logistic regression analyses were performed in different groups of participants, including three age groups (≥60 years old, 60–69 years old, and ≥70 years old) and those who had not yet received the booster dose. All independent variables were added to our multivariable models, and adjusted odds ratios (aORs) and 95% CIs were calculated via a backward stepwise method (P < 0.2). The Hosmer and Lemeshow test was used to assess the goodness of model fitting. All statistical analyses in this study were conducted using SPSS 26.0 (IBM SPSS Inc., NY, USA) and R 4.1.3 (AT&T Bell Lab., Auckland, New Zealand), and two-sided *P* < 0.05 was considered statistically significant.

## Results

### Participant characteristics

A total of 3,321 respondents were eventually included in our analyses (Table 1), and 1,097 (33.0%) were aged 70 or above. Among them, 1,731 (52.1%) were female, 926 (27.9%) lost their spouses, 1,987 (59.9%) did not have a high school degree, and more than 80% were satisfied with the government's response to COVID-19. A total of 9.7% of them had never been vaccinated, even with a single dose. Of all the participants, 82.8% (95% CI, 81.5–84.1%) were willing to receive COVID-19 vaccine booster shots (Table 1). People who were younger, lived with spouses, had a higher education level, or had a vaccination history were more likely to accept. Additionally, the more time spent on social media following content about COVID-19 and vaccines, the higher the vaccination willingness (*P*_trend_ < 0.05). Significant differences in vaccine acceptance were not found between groups stratified by region, sex, occupation, and economic condition (all *P* > 0.05).

**Table 1:** Acceptance of a COVID-19 vaccine booster dose among older adults in China by demographic characteristics (*n* = 3,321).

**Characteristics**	**Number (%)**	**Acceptance of a COVID-19 vaccination booster dose**		***P***
		**Yes (%)**	**95% CI**	
**Sociodemographic characteristics**				
**Region^a^**				0.96
Eastern	1,443 (43.5)	1,195 (82.8)	80.8–84.7	
Central	1,016 (30.6)	839 (82.6)	80.2–84.8	
Western	862 (26.0)	716 (83.1)	80.4–85.5	
**Age group (years)**				< 0.05^*^
60–64	982 (29.6)	850 (86.6)	84.3–88.6	
65–69	1,242 (37.4)	1,057 (85.1)	83.0–87.0	
≥70	1,097 (33.0)	843 (76.8)	74.3–79.3	
**Sex**				0.76
Female	1,731 (52.1)	1,430 (82.6)	80.8–84.3	
Male	1,590 (47.9)	1,320 (83.0)	81.1–84.8	
**Marital status ^b^**				< 0.05^*^
Married	2,290 (69.0)	1,941 (84.8)	83.2–86.2	
Widowed	926 (27.9)	723 (78.1)	75.3–80.7	
Others	105 (3.2)	86 (81.9)	73.7–88.4	
**Education^c^**				< 0.05^*^
Beyond high school	499 (15.0)	428 (85.8)	82.5–88.6	
High school	835 (25.1)	715 (85.6)	83.1–87.9	
Junior high school	905 (27.3)	751 (83.0)	80.4–85.3	
Primary and below	1,082 (32.6)	856 (79.1)	76.6–81.5	
**Occupation**				0.07
Individual household	1,050 (31.6)	886 (84.4)	82.1–86.5	
Employees of enterprise/public institutions	480 (14.5)	408 (85.0)	81.6–88.0	
Peasant	1,444 (43.5)	1,179 (81.6)	79.6–83.6	
Others	347 (10.4)	277 (79.8)	75.4–83.8	
**Monthly household income per capita (RMB)**				0.30
≤ 1,500	503 (15.1)	402 (79.9)	76.3–83.2	
1,501–1,000	719 (21.7)	596 (82.9)	80.0–85.5	
3,001–5,000	1,024 (30.8)	845 (82.5)	80.1–84.8	
5,001–10,000	791 (23.8)	667 (84.3)	81.7–86.7	
>10,000	284 (8.6)	240 (84.5)	80.0–88.4	
**Time spent on social media (min)**				< 0.05^*^
< 15	952 (28.7)	721 (75.7)	72.9–78.4	
15–30	1,390 (41.9)	1,184 (85.2)	83.2–87.0	
31–60	736 (22.2)	642 (87.2)	84.7–89.5	
>60	243 (7.3)	203 (83.5)	78.5–87.8	
**Satisfaction with the government's response to COVID-19**				< 0.05^*^
Not satisfied	124 (3.7)	83 (66.9)	58.3–74.8	
Neutral attitude	503 (15.1)	391 (77.7)	73.9–81.2	
Satisfied	1,353 (40.7)	1,116 (82.5)	80.4–84.4	
Very satisfied	1,341 (40.4)	1,160 (86.5)	84.6–88.3	
**Health status**				
**History of chronic disease**				< 0.05^*^
None	571 (17.2)	483 (84.6)	81.5–87.4	
≤ 1 year	284 (8.6)	235 (82.7)	78.0–86.8	
1–5 years	1,513 (45.6)	1,282 (84.7)	82.9–86.5	
6–10 years	651 (19.6)	516 (79.3)	76.0–82.2	
>10 years	302 (9.1)	234 (77.5)	72.5–81.9	
**History of COVID-19 vaccination** ^d^				< 0.05^*^
No vaccination	322 (9.7)	110 (34.2)	29.1–39.5	
Single dose	230 (6.9)	146 (63.5)	57.1–69.5	
Full vaccination	1,002 (30.2)	759 (75.7)	73.0–78.3	
Booster dose	1,767 (53.2)	1,735 (98.2)	97.5–98.7	
**Knowledge factors**				
**Total knowledge score on COVID-19**				< 0.05^*^
Low (score 0–6)	120 (3.6)	81 (67.5)	58.8–75.4	
Moderate (score 7–13)	1,762 (53.1)	1,404 (79.7)	77.8–81.5	
High (score 14–19)	1,439 (43.3)	1,265 (87.9)	86.1–89.5	
**Total knowledge score on COVID-19 vaccination**				< 0.05^*^
Low (score 0–1)	247 (7.4)	188 (76.1)	70.5–81.1	
Moderate (score 2–3)	2,461 (74.1)	2,040 (82.9)	81.4–84.3	
High (score 4)	613 (18.5)	522 (85.2)	82.2–87.8	
**Total**	3,321 (100)	2,750 (82.8)	81.5–84.1	

*P* values denote comparison of vaccine acceptance in different groups of characteristics (two-tailed chi-squared test).

* *P* < 0.05.

a “Eastern region (11 provinces)” included Beijing, Tianjin, Hebei province, Liaoning province, Shanghai, Jiangsu province, Zhejiang province, Fujian province, Shandong province, Guangdong province, and Hainan province; “Central region (8 provinces)” included Shanxi province, Jilin province, Heilongjiang province, Anhui province, Jiangxi province, Henan province, Hubei province, and Hunan province; “Western region (12 provinces)” included the Nei Monggol Autonomous Region, Chongqing, Guangxi province, Sichuan province, Guizhou province, Yunnan province, Tibet, Shaanxi province, Gansu province, Qinghai province, Ningxia province, and the Xinjiang Uygur Autonomous Region.

b “Married” referred to married elderly people whose spouses were still alive.

c “High school” included high school education and technical secondary school education.

d “No vaccination” referred to people who were not vaccinated at all; “Single dose” meant only received one dose of inactivated vaccine; “Full vaccination” referred to complete vaccination without a booster dose.

### Comparison of COVID-19 vaccine booster dose acceptance based on HBM

Concerns about the susceptibility of themselves and family members to SARS-CoV-2 infection indicated a higher acceptance rate of the booster dose among the older population (*P* < 0.05) (Table 2). On the “perceived severity” dimension, group differences between participants who agreed that their infection would cause severe illness or put their family at risk and participants who disagreed were not statistically significant (*P* > 0.05). Elderly people who believed in the safety and effectiveness of COVID-19 booster shots showed higher acceptance rates of 83.20% (81.90–84.50%) and 83.80% (82.40–85.10%), respectively. Similar results were exhibited for the dimension of the perceived benefit of the COVID-19 booster shot. Based on the analysis of five dimensions of the HBM, the higher the perception of susceptibility, benefit, and cues to action, the higher the vaccination acceptance rate among the elderly (all *P*_trend_ < 0.05).

**Table 2:** Comparison of the acceptance rate of the COVID-19 vaccine booster dose based on the health belief model among the elderly population in China (*n* = 3,321).

**Dimensions of health belief model**	**Item**	**Response^a^**	**Number (%)**	**Acceptance of a COVID-19 vaccine booster dose**		***P***
				***n* (%)**	**95% CI**	
Perceived susceptibility	Are you concerned about getting COVID-19?	Not concerned	867 (26.1)	671 (77.40)	74.50–80.10	< 0.05^*^
		Concerned	2,454 (73.9)	2,079 (84.70)	83.30–86.10	
	Are you worried about your family contracting COVID-19?	Not concerned	486 (14.6)	387 (79.60)	75.90–83.00	< 0.05^*^
		Concerned	2,835 (85.4)	2,363 (83.40)	81.90–84.70	
Perceived severity	People who get COVID-19 are more likely to get severe illness	Disagree	1,861 (56.0)	1,561 (83.90)	82.20–85.50	0.06
		Agree	1,460 (44.0)	1,189 (81.40)	79.40–83.40	
	When you get COVID-19, your family's health may be at risk	Disagree	506 (15.2)	410 (81.00)	77.40–84.30	0.25
		Agree	2,815 (84.8)	2,340 (83.10)	81.70–84.50	
Perceived barriers	A booster dose of COVID-19 vaccine can cause infection	Disagree	2,998 (90.3)	2,482 (82.80)	81.40–84.10	0.93
		Agree	323 (9.7)	268 (83.00)	78.60–86.80	
	It is not safe to get a booster dose against COVID-19	Disagree	3,183 (95.8)	2,649 (83.20)	81.90–84.50	< 0.05^*^
		Agree	138 (4.2)	101 (73.20)	65.40–80.00	
	It is not effective to get a booster dose against COVID-19	Disagree	3,026 (91.1)	2,535 (83.80)	82.40–85.10	< 0.05^*^
		Agree	295 (8.9)	215 (72.90)	67.60–77.70	
Perceived benefit	It is good to strengthen your health with COVID-19 vaccination	Disagree	957 (28.8)	658 (68.80)	65.80–71.60	< 0.05^*^
		Agree	2,364 (71.2)	2,092 (88.50)	87.20–89.70	
	It is good for family health when vaccinating a booster dose	Disagree	872 (26.3)	641 (73.50)	70.50–76.40	< 0.05^*^
		Agree	2,449 (73.7)	2,109 (86.10)	84.70–87.40	
	A booster dose can provide better protection against COVID-19	Disagree	570 (17.2)	409 (71.80)	68.00–75.30	< 0.05^*^
		Agree	2,751 (82.8)	2,341 (85.10)	83.70–86.40	
Cues to action	If your doctor/nurse recommends that you get a booster dose against COVID-19, you will choose it	Disagree	779 (23.5)	543 (69.70)	66.40–72.90	< 0.05^*^
		Agree	2,542 (76.5)	2,207 (86.80)	85.50–88.10	
	If your family recommends you to get a booster dose, you will take it	Disagree	646 (19.5)	423 (65.50)	61.80–69.10	< 0.05^*^
		Agree	2,675 (80.5)	2,327 (87.00)	85.70–88.20	
	If the community recommends that you get a booster dose against COVID-19, you will choose it	Disagree	1,289 (38.8)	928 (72.00)	69.50–74.40	< 0.05^*^
		Agree	2,032 (61.2)	1,822 (89.70)	88.30–90.90	

*P* values denote comparison of vaccine acceptance in different groups of characteristics (two-tailed chi-squared test).

* *P* < 0.05.

a “Very concerned” and “concerned” were combined to be “concerned”; and “disagree/not sure” were combined to be “disagree.”

### Factors related to COVID-19 vaccine booster dose acceptance

For all 3,321 participants, our multivariable logistic regression indicated that the main factors related to COVID-19 vaccine booster dose acceptance were occupation, time spent on social media, and higher knowledge scores on COVID-19 and vaccines (Table 3). Vaccination history (single dose, aOR = 3.45, 95% CI, 2.33–5.10; full vaccination, aOR = 14.94, 95% CI, 11.16–20.01) significantly promoted vaccination. In addition, older adults with a low level of perceived barriers (low, aOR = 1.86, 95% CI, 1.03–3.38), a high level of perceived benefit (high, aOR = 2.31, 95% CI, 1.38–3.87), and higher cues to action (moderate, aOR = 2.22, 95% CI, 1.39–3.56; high, aOR = 5.46, 95% CI, 3.44–8.67) were more likely to accept the booster shots. However, occupation and knowledge scores for COVID-19 were no longer considered as the influential factors for participants aged 60–69 (Table 3). For individuals 70 years of age or older, high perceived susceptibility (high, aOR = 2.07, 95% CI, 1.15–3.73) and cues to action (high, aOR = 5.29, 95% CI, 2.40–11.68) indicated a higher vaccination willingness. The results of univariable logistic regression models of these three groups are shown in Supplementary Table S2.

**Table 3:** Multivariable logistic regression of the factors associated with the acceptance of a COVID-19 vaccine booster dose among older people (*n* = 3,321).

**Characteristics**	**Model 1 (total**, ***n*** = **3,321)**		**Model 2 (60–69 years**, ***n*** = **2,224)**		**Model 3 (**≥**70 years**, ***n*** = **1,097)**	
	**aOR (95% CI)**	***P***	**aOR (95% CI)**	***P***	**aOR (95% CI)**	***P***
**Occupation**						
Others	0.67 (0.47–0.97)	< 0.05^*^			0.72 (0.39–1.34)	0.30
Individual household	0.81 (0.62–1.07)	0.14			0.59 (0.38–0.93)	< 0.05^*^
Employees of enterprise/public institutions	0.86 (0.61–1.21)	0.38			0.51 (0.28–0.95)	< 0.05^*^
Peasant	1 (reference)				1 (reference)	
**Time spent on social media (minutes)**						
< 15	1 (reference)		1 (reference)		1 (reference)	
15–30	1.47 (1.13–1.90)	< 0.05^*^	1.43 (1.03–2.00)	< 0.05^*^	1.38 (0.91–2.09)	0.13
31–60	1.86 (1.35–2.58)	< 0.05^*^	1.84 (1.24–2.74)	< 0.05^*^	1.49 (0.85–2.62)	0.17
>60	1.49 (0.94–2.37)	0.09	1.17 (0.67–2.02)	0.59	2.21 (0.92–5.32)	0.08
**History of chronic disease**						
No	1 (reference)					
≤ 1 year	0.95 (0.60–1.51)	0.82				
1–5 years	1.19 (0.87–1.64)	0.28				
6–10 years	0.82 (0.57–1.17)	0.26				
>10 years	0.98 (0.63–1.51)	0.92				
**History of COVID-19 vaccination^a^**						
No vaccination	1 (reference)		1 (reference)		1 (reference)	
Single dose	3.45 (2.33–5.10)	< 0.05^*^	4.96 (2.82–8.72)	< 0.05^*^	2.55 (1.45–4.49)	< 0.05^*^
Full vaccination	14.94 (11.16–20.01)	< 0.05^*^	14.95 (9.95–22.45)	< 0.05^*^	16.33 (10.57–25.23)	< 0.05^*^
**Knowledge factors**						
**Total knowledge score on COVID-19**						
Low (score 0–6)	1 (reference)		1 (reference)		1 (reference)	
Moderate (score 7–13)	1.21 (0.74–1.99)	0.45	1.43 (0.73–2.81)	0.30	0.91 (4.34–1.89)	0.79
High (score 14–19)	1.71 (1.01–2.88)	< 0.05^*^	1.87 (0.93–3.76)	0.08	1.38 (0.63–0.02)	0.42
**Total knowledge score on COVID-19 vaccination**						
Low (score 0–1)	1 (reference)		1 (reference)			
Moderate (score 2–3)	0.64 (0.43–0.96)	< 0.05^*^	0.53 (0.21–0.91)	< 0.05^*^		
High (score 4)	0.65 (0.40–1.05)	< 0.05^*^	0.49 (0.26–0.91)	< 0.05^*^		
**Health belief factors**						
**Perceived susceptibility**						
Low (score 2–3)					1 (reference)	
Moderate (score 4–5)					1.32 (0.87–1.98)	0.19
High (score 6)					2.07 (1.15–3.73)	< 0.05^*^
**Perceived barriers**						
Low (score 3–4)	1.86 (1.03–3.38)	< 0.05^*^	2.31 (1.15–4.61)	< 0.05^*^	1.29 (0.39–4.24)	0.68
Moderate (score 5–7)	0.81 (0.44–1.47)	0.48	1.01 (0.51–2.03)	0.97	0.51 (0.15–1.69)	0.27
High (score 8–9)	1 (reference)		1 (reference)		1 (reference)	
**Perceived benefit**						
Low (score 3–5)	1 (reference)		1 (reference)		1 (reference)	
Moderate (score 6–7)	1.17 (0.69–1.97)	0.57	1.51 (0.78–2.90)	0.22	0.66 (0.27–1.60)	0.35
High (score 8–9)	2.31 (1.38–3.87)	< 0.05^*^	2.80 (1.47–5.33)	< 0.05^*^	1.51 (0.64–3.53)	0.35
**Cues to action**						
Low (score 3–5)	1 (reference)		1 (reference)		1 (reference)	
Moderate (score 6–7)	2.22 (1.39–3.56)	< 0.05^*^	2.26 (1.26–4.04)	< 0.05^*^	2.06 (0.91–4.66)	0.08
High (score 8–9)	5.46 (3.44–8.67)	< 0.05^*^	5.55 (3.13–9.85)	< 0.05^*^	5.29 (2.40–11.68)	< 0.05^*^

aOR, adjusted odds ratio of multivariable logistic regression model via a backward stepwise method (*P* < 0.2).

* *P* < 0.05.

a “No vaccination” referred to people who were not vaccinated at all; “single dose” referred to only one dose of inactivated vaccine; “full vaccination” referred to complete vaccination, including people who have received a booster dose.

**Figure 1:**
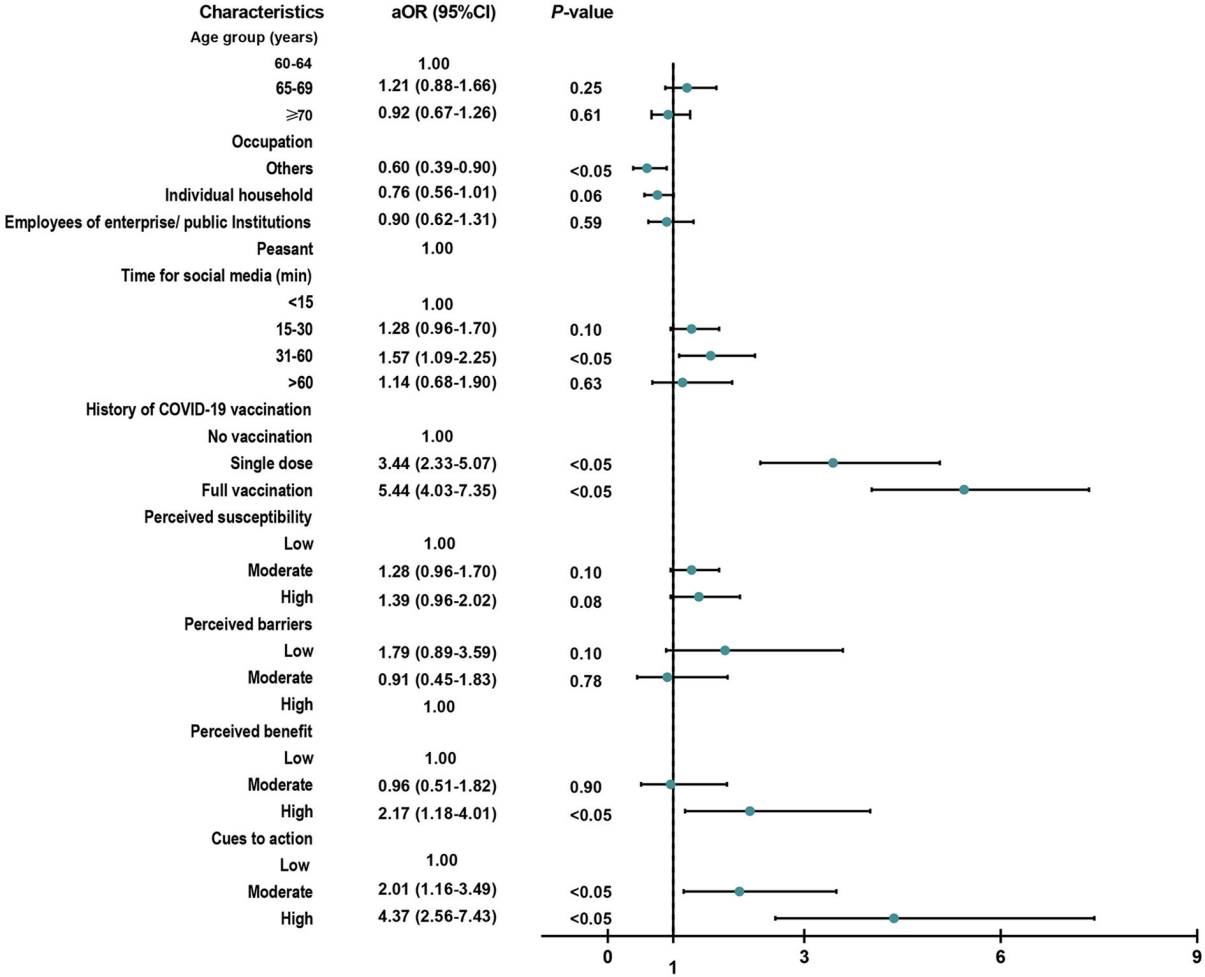
Factors related to vaccination acceptance among 1,554 participants who did not receive booster shots. aOR, adjusted odds ratio of multivariable logistic regression model *via* a backward stepwise method (*P* < 0.2).

Among the 1,554 participants who did not receive booster shots, 65.3% showed a willingness to receive the booster vaccination. A higher level of perceived benefit (high, aOR = 2.17, 95% CI, 1.18–4.01) and cues to action (moderate, aOR = 2.01, 95% CI, 1.16–3.49; high, aOR = 4.37, 95% CI, 2.56–7.43) indicated a higher willingness to receive a booster dose among those elderly people. Additionally, occupation, time spent on social media, and vaccination history could also affect the attitudes of elderly people toward booster vaccination (Figure 1).

### Reasons for COVID-19 booster dose vaccine hesitancy stratified by age

**Figure 2:**
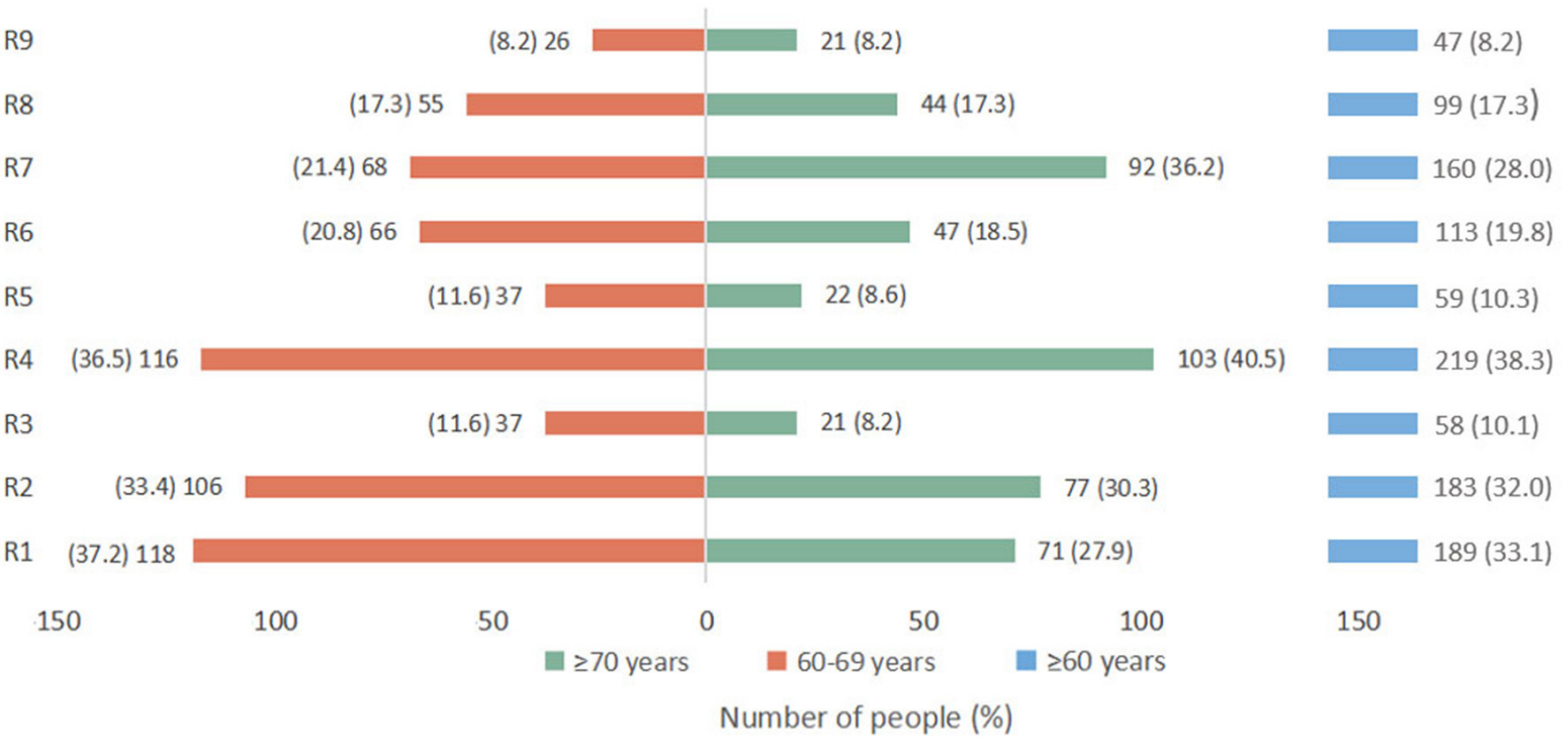
Reasons for responding “no” or “not sure” when asked about willingness to accept the COVID-19 vaccine booster dose (*n* = 571). “R1,” one or two doses of COVID-19 vaccines are already sufficient and a third dose is unnecessary; “R2,” the safety of the COVID-19 vaccine is not clear; “R3,” the efficacy of the COVID-19 vaccine is not clear; “R4,” severe illness and ineligibility for vaccination; “R5,” believing that they are healthy enough to fight COVID-19; “R6,” COVID-19 in China is well under control and there is no need to vaccinate; “R7,” limited movement; “R8,” believing that the vaccination process is complicated and time consuming; “R9,” other reasons.

**Figure 3:**
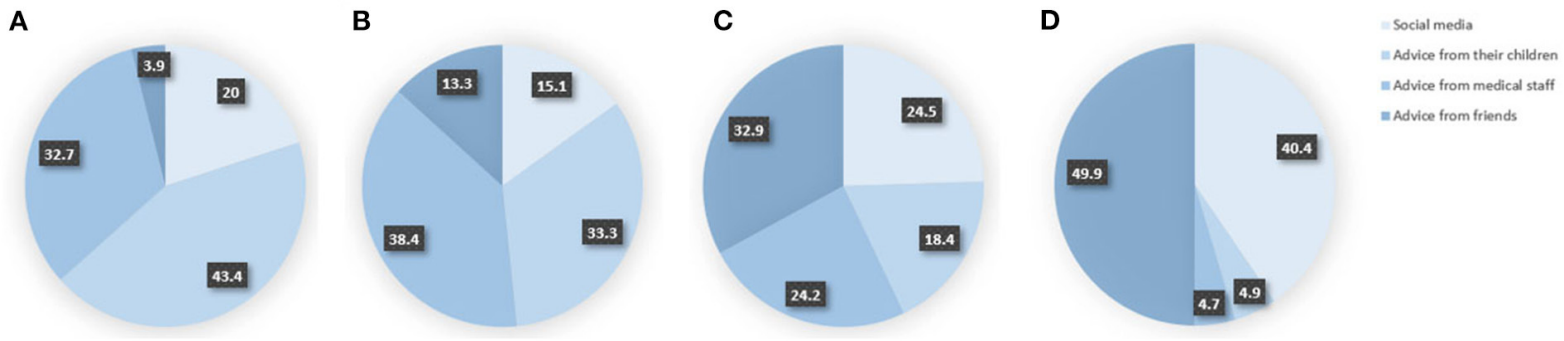
Ranking of the influence degree of four factors on the willingness of the elderly to receive the booster vaccination. **(A)** The most important, **(B)** the second most important, **(C)** the third most important, and **(D)** the least important.

Among the 571 (17.2%) participants who were reluctant or refused to receive the booster dose, nearly one-third were still unaware of the importance of booster shots, believing that it was unnecessary to receive a booster dose as they had already received one or two doses of the initial vaccination (Figure 2). A total of 38.3, 32, and 28% hesitated due to their concerns about contraindications, vaccine safety, and limited movement, respectively. Worrying about whether they were eligible for booster shots due to their existing diseases was a non-negligible reason for people aged 70 years or above. When ranking the four factors (social media, and advice from children, medical staff, and friends) that influence the elderly's vaccination willingness, 43.4% agreed that advice from children was the most important factor, while 32.7% supported advice from medical staff (Figure 3).

## Discussion

As a high-risk population with poor prognosis after infection, older adults are the key population for follow-up booster vaccination. We found that, of the 3,321 participants recruited, 82.8% (95% CI, 81.5–84.1%) were willing to receive COVID-19 vaccine booster shots. Concerns about contraindications, vaccine safety, and limited movement were the main reasons for vaccine hesitancy. Factors related to vaccine acceptance rate were occupation, time spent on social media, vaccination history, knowledge factors, and perception of barriers and benefit, as well as action cues. For the over 70s, increasing their awareness of susceptibility could be a better gateway for improving vaccination acceptance. Given the current global epidemic situation and the prevalence of highly infectious variants, it is likely that the subsequent second and third booster shots or vaccines against specific variants will soon be administered globally (40). Older adults have been prioritized for protection against severe illness and death through vaccination during this pandemic. Accordingly, our findings can help agencies develop targeted strategies to provide longer lasting immunity and greater protection against the evolving SARS-CoV-2 variants, which is important for the health of the elderly and the population as a whole.

Based on our study, 82.8% of 3,321 participants recruited were willing to receive COVID-19 vaccine booster shots. As the high-risk group for severe illness and death after SARS-CoV-2 infection, 87.6 million people (33.2%) aged 60 years and above in China have yet to receive booster shots as of August 10, 2022 (16). To date, very few studies have specifically focused on intentions to receive booster shots and the influencing factors among older adults, so our results can only be compared with specific subgroups of several existing studies (20, 39, 41). A large-scale nationwide survey conducted in China found that only 10.56% (30/284) of older adults (≥60 years old) were hesitant about receiving a booster shot (38), whereas in Jordan and Bangladesh (≥50 years old), the rates were 35.0 and 44.6%, respectively (20, 41). In addition, time spent on social media tracking news about COVID-19 and vaccines was also associated with vaccination acceptance (42). Our study also reflected the positive effect of vaccination history on receiving the booster shots, which was consistent with previous studies (19, 21, 39). Another surprising result was that, among older adults, the higher the knowledge scores on COVID-19 vaccines, the lower the willingness to take booster shots. More efforts are needed to regulate social media content and filter out misinformation (42, 43). The role of official social media in disseminating health information should be strengthened (42, 43).

Moreover, the HBM attempted to explain and predict the intention and behavior of the elderly with regard to receiving the COVID-19 vaccine booster shot from five dimensions (19–21). According to our multivariable analysis, people with low perceived barriers (model 1, aOR = 1.86, 95% CI, 1.03–3.38; model 2, aOR = 2.31, 95% CI, 1.15–4.61) and high perceived benefit (model 1, aOR = 2.31, 95% CI, 1.38–3.87; model 2, aOR = 2.80, 95% CI, 1.47–5.33) showed higher vaccination intention, which was generally consistent with previous studies (30, 44–46). However, for the perception of severity, none of our models found an association with vaccination willingness (all *P* > 0.05). Multiple studies have shown that higher perceived severity is negatively associated with vaccine hesitancy (38, 46, 47). For the over 70s, improving their awareness of susceptibility to SARS-CoV-2 variants could be a better gateway for making them more receptive to the vaccine. Of all the models, high action cues proved to have the most significant effect on vaccination willingness (all *P* < 0.05), which highlighted the importance and advantages of families and healthcare workers encouraging vaccination for eligible older persons. Similar results can also be verified (13, 19). Although the overall acceptance of COVID-19 booster shots among the elderly (≥60 years old) in China is relatively higher than that in other countries, efforts should also be made to remove barriers to vaccination given the large population base.

According to our results, nearly one-third of the elderly were still unaware of the importance of booster shots, believing a booster dose was unnecessary. According to a report on the fifth wave of COVID-19 in Hong Kong, China, people aged 60 years or more accounted for 96% of all deaths, and the mortality rate was 0.7% for those who were unvaccinated and 0.02% for those who had received two or three doses (14). Another concern is that the overall levels of effective antibodies produced by older people after vaccination are relatively lower than those produced by younger people (10, 17). We found that for those over 70 years of age, worrying about whether they were eligible for the booster shots due to their pre-existing severe or chronic diseases in acute phase was a non-negligible reason (40.5%, 103/254). As risk factors for SARS-CoV-2 infection and poor prognosis, comorbidities such as hypertension, diabetes, cardiovascular disease, and chronic respiratory disease are more common in older people (9, 10). Advanced age was also a risk factor for the disease (9, 10). Patients with well-controlled chronic diseases are generally considered eligible for vaccination (48). People with other medical conditions should consult health professionals in detail to determine whether they are contraindicated for vaccination (48). Regarding safety and effectiveness, data from the US, Germany, and South Korea all confirmed that prioritizing the most elderly for COVID-19 vaccination could save the most lives and surprisingly maximize remaining life expectancy (49).

We must recognize a practical problem, that is, the longer it takes to achieve herd immunity, the longer it will take for normal economic development to resume (12, 18, 50) and more people will likely face more complex situations. Our findings will help the assessment of older adults' attitudes toward the booster dose and exploration of the associated factors influencing their vaccination behavior, which could provide theoretical and practical implications for subsequent immunization strategies in older populations. However, this study also had some limitations. First, as with other online surveys, selection bias may exist. The accessibility of online surveys for the elderly was an inescapable problem as questionnaires were answered only by internet users. However, given the current global epidemic and the endless stream of variants, vaccine hesitation, especially in the elderly population, is a serious threat to people's lives and health. Considering the current requirements of epidemic prevention and control, we chose to conduct an online survey *via* a specialized data science company with a personal information database. We set up tips at the end of each question, repeatedly emphasizing that the subjective questions were supposed to be answered by the elderly independently. Second, people's acceptance of the booster dose was measured only by using the self-report questionnaire, and we were unable to assess it *via* a standard scale. In addition, our results need to be extrapolated with caution because of the differences in countries and theoretical models, and we also hope that large-scale offline surveys with more participants will be implemented as soon as feasible.

## Conclusions

In this nationwide cross-sectional study, 82.8% of recruited older adults were willing to receive the COVID-19 vaccine booster dose. Acceptance behaviors were closely related to occupation, time spent on social media, vaccination history, knowledge factors, perception of barriers and benefit, and action cues. Concerns about contraindications, vaccine safety, and limited movement were the main reasons for vaccine hesitancy. In the current situation, promoting vaccination coverage of valid immunity among the elderly population is quite urgent, not only to prevent infection, severe illness, and death caused by emerging variants but also to reduce the huge disease and economic burden caused by the long-term sequelae of COVID-19.

## Data availability statement

The datasets presented in this article are not readily available because the data will be further used in the following articles and research, and are not publicly available at this time. Requests to access the datasets should be directed to JL, jueliu@bjmu.edu.cn.

## Ethics statement

This study met the requirements of the Declaration of Helsinki and was approved by the Institutional Review Board of Peking University (IRB00001052-21126). The patients/participants provided their written informed consent to participate in this study.

## Author contributions

Conceptualization: CQ, MD, and WY. Methodology and analysis, visualization, and writing—original draft preparation: CQ. Review and editing: LT, WY, MD, QL, and ML. Supervision: JL. All authors have read and agreed to the published version of the manuscript.
