# Supplementary material for: Acceptance of the COVID-19 vaccine booster dose and associated factors among the elderly in China based on the health belief model (HBM): A national cross-sectional study
Source: Front Public Health. 2022 Dec 15;10:986916. doi: 10.3389/fpubh.2022.986916 (PMC9797829; doi:10.3389/fpubh.2022.986916)
Supplement: Supplementary file 1 [file Data_Sheet_1.docx]

**Supplemental file** 1

**Table S1** Collection of valid questionnaires by region in mainland China

| **Regions** | **Number** | **Proportion** |
| --- | --- | --- |
| Guangdong Province | 190 | 5.72% |
| Shandong Province | 260 | 7.83% |
| Henan Province | 223 | 6.71% |
| Jiangsu Province | 230 | 6.93% |
| Sichuan Province | 224 | 6.74% |
| Hebei Province | 190 | 5.72% |
| Hunan Province | 161 | 4.85% |
| Zhejiang Province | 149 | 4.49% |
| Anhui Province | 140 | 4.22% |
| Hubei Province | 145 | 4.37% |
| Guangxi Province | 107 | 3.22% |
| Jiangxi Province | 95 | 2.86% |
| Liaoning Province | 140 | 4.22% |
| Fujian Province | 85 | 2.56% |
| Shaanxi Province | 96 | 2.89% |
| Guizhou Province | 76 | 2.29% |
| Shanxi Province | 90 | 2.71% |
| Chongqing | 89 | 2.68% |
| Heilongjiang Province | 92 | 2.77% |
| Yunnan Province | 90 | 2.71% |
| the Nei Monggol Autonomous Region | 61 | 1.84% |
| Jilin Province | 70 | 2.11% |
| Shanghai | 80 | 2.41% |
| Gansu Province | 54 | 1.63% |
| Beijing | 60 | 1.81% |
| the Xinjiang Uygur Autonomous Region | 36 | 1.08% |
| Tianjin | 39 | 1.17% |
| Hainan Province | 20 | 0.60% |
| Ningxia Province | 15 | 0.45% |
| Tibet | 4 | 0.12% |
| Qinghai Province | 10 | 0.30% |

**Supplemental file 2.**

**A questionnaire about the acceptance of a booster dose of COVID-19 vaccine among older adults**

Thank you very much for participating in this survey. The main purpose of this questionnaire is to understand the willingness and influencing factors of a booster dose of COVID-19 vaccine in China. Please answer according to your true thoughts or actual situation.

This survey is only used for academic research and will be Not involved commercial use or privacy disclosure. This is an anonymous survey and no right or wrong answer. Please fill in the answer according to your actual situation. Thank you very much for taking up your precious time!

**Ⅰ. Sociodemographic characteristics and health status**

1. Age: ___________

2: What is your gender? [Single choice] *

| ○ man |
| --- |
| ○ woman |

3. What is your current marital status? [Single choice] *

○Married

○Widowed

○Others

4. What is your level of education? [Single choice] *

| ○Beyond high school |
| --- |
| ○High school |
| ○Junior high school |
| ○Primary and below |

5. What is your occupation before retirement? [Single choice] *

| ○Individual household |
| --- |
| ○Employees of enterprise/ public institutions |
| ○Peasant |
| ○Others |

6. Which average monthly income (RMB) group do you belong to? [Single choice] *

| ○≤1,500 |
| --- |
| ○1,501-1,000 |
| ○3,001-5,000 |
| ○5,001-10,000 |
| ○>10,000 |

7. Do you have any chronic disease (such as cardiovascular disease, cancer, diabetes, chronic respiratory disease, etc.)? [Single choice] *

| ○ No |
| --- |
| ○≤1 year |
| ○1-5 years |
| ○6-10years |
| ○>10years |

8. Have you received the COVID-19 vaccine? [single choice] *

| ○ Yes, single dose |
| --- |
| ○ Yes, full vaccination |
| ○ Yes, booster dose |
| ○ No |

**II. Acceptance for a booster dose of COVID-19 vaccine**

9. Are you willing to receive a booster dose of COVID-19 vaccination if available? [single choice] *

| ○ Yes |
| --- |
| ○ No  ○ not sure |

10. Which of the following reasons are you not willing or sure to get a booster dose of COVID-19 vaccine? [Multiple optional] *

| □ One or two doses of COVID-19 vaccines is already sufficient and a third dose is unnecessary |
| --- |
| □ The safety of the COVID-19 vaccine is not clear |
| □ The efficacy of the COVID-19 vaccine is not clear |
| □ Severe illness and ineligibility for vaccination |
| □ Believing that they are healthy enough to fight COVID-19 |
| □ COVID-19 in China is under great control and there is no need to vaccinate |
| □ Limited movements |
| □ Believing that the vaccination process is complicated and time-wasting |
| Other reasons |

11. To what extent do the following approaches affect your willingness to receive a booster shot? Order them from most important to least important

| ○ Social media |
| --- |
| ○ Advice from their children |
| ○Advice from medical staff |
| ○Advice from friends |

12. Do you satisfied Not satisfied with the government’s response to COVID-19? [single choice] *

| ○Not satisfied |
| --- |
| ○Neutral attitude  ○Satisfied |
| ○Very satisfied |

**III. Knowledge of COVID-19 and vaccines**

13. Which of the following do you think is the source of infection for COVID-19? [Single choice] *

| ○ Patients |
| --- |
| ○ the asymptomatic |
| ○ Both |
| ○ Not clear |

14. Which of the following do you think are common symptoms of COVID-19? [Multiple optional] *

| □ Fever |
| --- |
| □ Diarrhea |
| □ Lack of power |
| □ Cough |
| □ Loss of sense of smell or taste |
| □ Chest pain |
| □ Not clear |

15. Which of the following do you think are ways to prevent COVID-19? [Multiple optional] *

| □ Cover your mouth and nose with tissue or towel when coughing or sneezing |
| --- |
| □ Balanced diet |
| □ Get enough exercise and rest |
| □ Wearing a mask, and wash hands frequently |
| □ Not clear |

16. Who do you think is at high risk for severe/critical COVID-19 [Multiple optional] *

| □ Older than 65 years |
| --- |
| □ With chronic disease |
| □ Heavy smoker |
| □ Third trimester and perinatal women |
| □ Immune deficiency |
| □ Obesity ( BMI≥30) |
| □ Not clear |

17. What do you think is the possible transmission route of COVID-19? [Multiple optional] *

| □ Droplet infection |
| --- |
| □ Contact infection |
| □ Aerosol transmission |
| □ Fecal-oral route of transmission |
| □ Not clear |

18. Do you think the following statement about COVID-19 is correct [Matrix Single choice] *

|  | Yes | Not sure | No |
| --- | --- | --- | --- |
| (1) People are generally susceptible to COVID-19. | ○ | ○ | ○ |
| (2) COVID-19 vaccination is free in China. | ○ | ○ | ○ |
| (3) At present, it is recommended that the booster dose vaccination should be complete can only be received 6 months after the whole vaccination. | ○ | ○ | ○ |
| (4) After vaccination, the protection decreases over time. | ○ | ○ | ○ |
| (5) There may be adverse reactions after receiving COVID-19 vaccine |  |  |  |

**IV.** **Health beliefs on COVID-19 and vaccination**

19. Do you have the following concerns? [Matrix Single choice] *

|  | Very Concerned | Concerned | Not Concerned |
| --- | --- | --- | --- |
| Are you concerned about getting COVID-19? | ○ | ○ | ○ |
| Are you concerned about family members getting COVID-19? | ○ | ○ | ○ |

20. Do you agree with this statement? [Matrix Single choice] *

|  | Agree | Not Sure | Disagree |
| --- | --- | --- | --- |
| (1) People who get COVID-19 are more likely to get severe illness. | ○ | ○ | ○ |
| (2) When you get COVID-19, your family's health may be at risk. | ○ | ○ | ○ |
| (3) A booster dose of COVID-19 vaccine can cause infection. | ○ | ○ | ○ |
| (4) It is not safe to get a booster dose against COVID-19. | ○ | ○ | ○ |
| (5) It is not effective to get a booster dose against COVID-19. | ○ | ○ | ○ |
| (6) It is good to strengthen your health with COVID-19 vaccination. | ○ | ○ | ○ |
| (7) It is good for family health when vaccinating a booster dose. | ○ | ○ | ○ |
| (8) A booster dose can provide better protection against COVID-19. | ○ | ○ | ○ |
| (9) If the doctor/nurse recommends me to get a booster shot of COVID-19 vaccine, I will choose to get it. |  |  |  |
| (10) If my family recommends me to get a booster shot of COVID-19 vaccine, I will choose to get it. |  |  |  |
| (11) If the community recommends me to get a booster shot of COVID-19 vaccine, I will choose to get it. |  |  |  |

**Supplemental file 3**

**Table S2** Univariable logistic regression of the factors associated with the acceptance of a booster dose of COVID-19 vaccine among the elderly (n=3,321)

| **Characteristics** | **Total**  **(n=3,321)** | |  | **60-69 years**  **(n=2,224)** | |  | **≥70 years**  **(n=1,097)** | |
| --- | --- | --- | --- | --- | --- | --- | --- | --- |
|  | Crude OR (95% CI) | ***P*** value |  | Crude OR (95% CI) | ***P*** value |  | Crude OR (95% CI) | ***P*** value |
| **Region**^a^ | | | | | |  |  |  |
| Eastern | 0.98 (0.79-1.23) | 0.88 |  | 1.00 (0.74-1.34) | 0.98 |  | 0.93 (0.65-1.31) | 0.67 |
| Central | 0.97 (0.76-1.23) | 0.78 |  | 1.06 (0.77-1.46) | 0.74 |  | 0.83 (0.58-1.21) | 0.33 |
| Western | 1 (reference) |  |  | 1 (reference) |  |  | 1 (reference) |  |
| **Sex** | | | | | |  |  |  |
| Female | 1 (reference) |  |  | 1 (reference) |  |  | 1 (reference) |  |
| Male | 1.03 (0.86-1.23) | 0.76 |  | 1.18 (0.93-1.49) | 0.18 |  | 0.88 (0.66-1.16) | 0.36 |
| **Marital status^b^** | | | | | |  |  |  |
| Others | 1.27 (0.76-2.14) | 0.37 |  | 1.04 (0.54-1.98) | 0.91 |  | 1.03 (0.40-2.67) | 0.95 |
| Married | 1.56 (1.29-1.89) | <0.05* |  | 1.24 (0.93-1.65) | 0.15 |  | 1.46 (1.10-1.94) | <0.05* |
| Widowed | 1 (reference) |  |  | 1 (reference) |  |  | 1 (reference) |  |
| **Education^c^** | | | | | |  |  |  |
| Beyond high school | 1.59 (1.19-2.13) | <0.05* |  | 1.46 (1.00-2.12) | <0.05* |  | 1.54 (0.96-2.49) | 0.08 |
| High school | 1.57 (1.23-2.01) | <0.05* |  | 1.53 (1.12-2.10) | <0.05* |  | 1.26 (0.85-1.87) | 0.26 |
| Junior high school | 1.29 (1.03-1.62) | <0.05* |  | 1.35 (0.99-1.84) | 0.06 |  | 1.03 (0.73-1.45) | 0.88 |
| Primary and below | 1 (reference) |  |  | 1 (reference) |  |  | 1 (reference) |  |
| **Occupation** | | | | | |  |  |  |
| Others | 0.89 (0.66-1.19) | 0.44 |  | 0.73 (0.50-1.06) | 0.10 |  | 1.12 (0.69-1.82) | 0.64 |
| Individual household | 1.21 (0.98-1.50) | 0.07 |  | 1.19 (0.90-1.58) | 0.22 |  | (1.05 (0.75-1.47) | 0.76 |
| Employees of enterprise/ public institutions | 1.27 (0.96-1.69) | 0.10 |  | 1.28 (0.89-1.85) | 0.19 |  | 0.97 (0.61-1.55) | 0.89 |
| Peasant | 1 (reference) |  |  | 1 (reference) |  |  | 1 (reference) |  |
| **Monthly household income per capita (RMB)** | | | | | |  |  |  |
| ≤1,500 | 1 (reference) |  |  | 1 (reference) |  |  | 1 (reference) |  |
| 1,501-1,000 | 1.22 (0.91-1.63) | 0.19 |  | 1.40 (0.93-2.11) | 0.10 |  | 0.93 (0.61-1.43) | 0.75 |
| 3,001-5,000 | 1.19 (0.90-1.56) | 0.22 |  | 1.07 (0.75-1.55) | 0.69 |  | 1.21 (0.80-1.84) | 0.37 |
| 5,001-10,000 | 1.35 (1.01-1.81) | 0.04 |  | 1.39 (0.93-2.06) | 0.11 |  | 1.19 (0.78-1.84) | 0.42 |
| >10,000 | 1.37 (0.93-2.02) | 0.11 |  | 1.21 (0.73-1.99) | 0.46 |  | 1.49 (0.79-2.79) | 0.21 |
| **Time for social media per day (minutes)** | | | | | |  |  |  |
| <15 | 1 (reference) |  |  | 1 (reference) |  |  | 1 (reference) |  |
| 15-30 | 1.84 (1.49-2.27) | <0.05* |  | 1.78 (1.34-2.36) | <0.05* |  | 1.69 (1.23-2.32) | <0.05* |
| 31-60 | 2.19 (1.68-2.84) | <0.05* |  | 2.10 (1.50-2.96) | <0.05* |  | 1.81 (1.18-2.77) | <0.05* |
| >60 | 1.63 (1.12-2.35) | <0.05* |  | 1.16 (0.75-1.82) | 0.51 |  | 2.74 (1.36-5.51) | <0.05* |
| **Satisfaction with the government’s response to COVID-19** | | | | | |  |  |  |
| Not satisfied | 1 (reference) |  |  | 1 (reference) |  |  | 1 (reference) |  |
| Neutral attitude | 1.73 (1.12-2.65) | <0.05* |  | 1.88 (1.09-3.23) | <0.05* |  | 1.58 (0.77-3.24) | 0.21 |
| Satisfied | 2.33 (1.56-3.47) | <0.05* |  | 2.34 (1.42-3.85) | <0.05* |  | 2.37 (1.21-4.67) | <0.05* |
| Very satisfied | 3.17 (2.11-4.75) | <0.05* |  | 3.60 (2.15-6.03) | <0.05* |  | 2.88 (1.46-5.68) | <0.05* |
| **History of chronic disease** | | | | | |  |  |  |
| No | 1 (reference) |  |  | 1 (reference) |  |  | 1 (reference) |  |
| ≤1 year | 0.87 (0.60-1.28) | 0.49 |  | 0.81 (0.49-1.34) | 0.41 |  | 1.03 (0.57-1.88) | 0.92 |
| 1-5 years | 1.01 (0.77-1.32) | 0.94 |  | 0.84 (0.59-1.20) | 0.34 |  | 1.36 (0.89-2.08) | 0.15 |
| 6-10 years | 0.70 (0.52-0.94) | <0.05* |  | 0.59 (0.40-0.88) | <0.05* |  | 0.99 (0.63-1.57) | 0.98 |
| >10 years | 0.63 (0.44-0.89) | <0.05* |  | 0.55 (0.33-0.93) | <0.05* |  | 0.98 (0.60-1.62) | 0.95 |
| **History of COVID-19 vaccination**^f^ | | | | | |  |  |  |
| No vaccination | 1 (reference) |  |  | 1 (reference) |  |  | 1 (reference) |  |
| Single dose | 3.35 (2.35-4.77) | <0.05* |  | 4.19 (2.52-6.97) | <0.05* |  | 2.57 (1.56-4.22) | <0.05* |
| Full vaccination | 17.48 (13.45-22.71) | <0.05* |  | 17.27 (11.91-25.02) | <0.05* |  | 16.43 (11.2-24.11) | <0.05* |
| **Total knowledge score on COVID-19** | | | | | |  |  |  |
| Low (score 0-6) | 1 (reference) |  |  | 1 (reference) |  |  | 1 (reference) |  |
| Moderate (score 7-13) | 1.89 (1.27-2.82) | <0.05* |  | 2.16 (1.23-3.78) | <0.05* |  | 2.57 (0.82-2.58) | 0.20 |
| High (score 14-19) | 3.50 (2.32-5.29) | <0.05* |  | 3.65 (2.05-6.47) | <0.05* |  | 16.43 (1.58-5.29) | <0.05* |
| **Total knowledge score on COVID-19 vaccination** | | | | | |  |  |  |
| Low (score 0-1) | 1 (reference) |  |  | 1 (reference) |  |  | 1 (reference) |  |
| Moderate (score 2-3) | 1.52 (1.12-2.08) | <0.05* |  | 1.33 (0.87-2.04) | 0.18 |  | 1.76 (1.10-2.82) | <0.05* |
| High (score 4) | 1.80 (1.25-2.60) | <0.05* |  | 1.50 (0.91-2.46) | 0.11 |  | 2.26 (1.28-3.98) | <0.05* |
| **Perceived susceptibility** | | | | | |  |  |  |
| Low | 1 (reference) |  |  | 1 (reference) |  |  | 1 (reference) |  |
| Moderate | 1.50 (1.22-1.85) | <0.05* |  | 1.41 (1.06-1.86) | <0.05* |  | 1.60 (1.17-2.19) | <0.05* |
| High | 1.39 (1.06-1.82) | <0.05* |  | 1.10 (0.77-1.55) | 0.61 |  | 1.88 (1.19-2.96) | <0.05* |
| **Perceived severity** | | | | | |  |  |  |
| Low | 1 (reference) |  |  | 1 (reference) |  |  | 1 (reference) |  |
| Moderate | 1.01 (0.72-1.41) | 0.95 |  | 1.23 (0.82-1.86) | 0.32 |  | 0.73 (0.41-1.30) | 0.29 |
| High | 0.91 (0.65-1.28) | 0.59 |  | 1.12 (074-1.69) | 0.61 |  | 0.68 (0.38-1.22) | 0.20 |
| **Perceived barriers** | | | | | |  |  |  |
| Low | 2.37 (1.44-3.89) | <0.05* |  | 3.19 (1.77-5.76) | <0.05* |  | 1.42 (0.56-3.59) | 0.46 |
| Moderate | 0.77 (0.47-1.27) | 0.31 |  | 1.02 (0.56-1.85) | 0.95 |  | 0.46 (0.18-1.16) | 0.10 |
| High | 1 (reference) |  |  | 1 (reference) |  |  | 1 (reference) |  |
| **Perceived benefit** | | | | | |  |  |  |
| Low | 1 (reference) |  |  | 1 (reference) |  |  | 1 (reference) |  |
| Moderate | 2.08 (1.37-3.18) | <0.05* |  | 2.12 (1.23-3.65) | <0.05* |  | 2.01 (1.01-3.98) | <0.05* |
| High | 5.40 (5.61-8.08) | <0.05* |  | 5.45 (3.24-9.15) | <0.05* |  | 5.28 (2.75-10.16) | <0.05* |
| **Cues to action** | | | | | |  |  |  |
| Low | 1 (reference) |  |  | 1 (reference) |  |  | 1 (reference) |  |
| Moderate | 3.09 (2.11-4.53) | <0.05* |  | 3.74 (2.31-6.07) | <0.05* |  | 2.31 (1.22-4.37) | <0.05* |
| High | 10.58 (7.30-15.35) | <0.05* |  | 11.50 (7.21-18.35) | <0.05* |  | 9.23 (4.96-17.15) | <0.05* |

**P*<0.05;

^a^ “Eastern region (11 provinces) ” included Beijing, Tianjin, Hebei province, Liaoning province, Shanghai, Jiangsu province, Zhejiang province, Fujian province, Shandong province, Guangdong province, and Hainan province; “Central region (8 provinces)” included Shanxi province, Jilin province, Heilongjiang province, Anhui province, Jiangxi province, Henan province, Hubei province, and Hunan province; “Western region (12 provinces)” included the Nei Monggol Autonomous Region, Chongqing, Guangxi province, Sichuan province, Guizhou province, Yunnan province, Tibet, Shaanxi province, Gansu province, Qinghai province, Ningxia province, and the Xinjiang Uygur Autonomous Region.^b^ “Married” referred to the married old people whose spouses were still alive;

^c^ “High school” included high school education and technical secondary school education;

^f^ “No vaccination” referred to people who were not vaccinated at all; “Single dose” meant only received one dose of inactivated vaccine; “Full vaccination” referred to complete vaccination, including people who have received a booster dose.
